# Effect of the COVID-19 pandemic on Vogt–Koyanagi–Harada disease

**DOI:** 10.1038/s41598-024-63957-1

**Published:** 2024-06-08

**Authors:** Tetsuya Muto, Masaaki Sakamoto, Shoichiro Kusuda, Yasuo Haruyama, Shigeki Machida, Shinichiro Imaizumi, Tetsuju Sekiryu

**Affiliations:** 1 Department of Ophthalmology, Dokkyo Medical University Saitama Medical Center, Koshigaya, 343-8555 Japan; 2 Department of Ophthalmology, Imaizumi Eye Hospital, 20-9 Domaecho, Koriyama, 963-8877 Japan; 3 Department of Ophthalmology, Fukushima Medical University, Fukushima, 960-1295 Japan; 4 Dokkyo Medical University Center for Research Cooperative and Support, Mibu, 321-0293 Japan

**Keywords:** COVID-19, Pandemics, Prevalence, Sex ratio, Visual acuity, Vogt–Koyanagi–Harada disease, Immunology, Medical research

## Abstract

To determine the disease prevalence rate and clinical characteristics of Vogt–Koyanagi–Harada (VKH) disease among new patients before and after the declaration of a state of emergency (April 7, 2020) in Japan. New patients and patients with newly diagnosed VKH disease were categorized into “Before” and “After” groups based on the initial visit. The prevalence rate, sex ratio, and age of patients newly diagnosed with VKH were compared between the groups. Best-corrected visual acuity (BCVA) and recurrence rates were compared among 59 patients observed for > 12 months after receiving pulse steroid therapy. For reference, we also examined the prevalence rate of patients newly diagnosed with acute angle closure (AAC) in the Before and After groups. The prevalence rates of VKH disease among newly diagnosed patients (*P* < 0.05) or patients with AAC (*P* < 0.001) were significantly higher in the After group. No significant differences in sex ratio or age of VKH disease were observed in both groups. BCVA and recurrence rates showed no significant differences. The COVID-19 pandemic increased the prevalence of VKH disease among new patients compared with that of AAC. However, the clinical features of VKH disease were unlikely affected by the COVID-19 pandemic.

## Introduction

The novel severe acute respiratory syndrome coronavirus 2 (SARS-CoV-2) was responsible for the severe coronavirus disease 2019 (COVID-19) outbreak that started in December 2019 in Wuhan, Hubei Province, China^1^. Because of the strong infectivity of SARS-CoV-2, the epidemic quickly spread around the world. To prevent additional COVID-19 expansion, lockdowns were performed worldwide. Activities were limited by these lockdowns, and life styles of many people changed. During this time, the number of people who did home carpentry in narrow homes increased, resulting in a reported increase in eye globe ruptures have been reported to be increased as a result^2,3^.

The government declared a state of emergency in seven urban areas in Japan on April 7 and extended it until May 25, 2020. In short, a state of emergency is not as strict as a lockdown, as was the case in many other countries. The government requested people to refrain from unnecessary outings, including going to restaurants, schools, and public facilities and traveling to avoid the “three Cs,” namely, closed spaces, crowded spaces, and close-contact settings. The state of emergency in Japan dramatically reversed domestic economic activity. Worldwide, COVID-19 expanded, with vaccination for COVID-19 being widely performed. As a result, ocular diseases accompanied by COVID-19 have been reported^4,5^. Furthermore, studies have reported great numbers of ocular diseases secondary to COVID-19 infection or COVID-19 vaccination^6–12^.

Several articles have reported the development or relapse of Vogt–Koyanagi–Harada (VKH) disease following COVID-19 infection or COVID-19 vaccination^12–19^. Accumulation of this evidence suggests a correlation between VKH disease and COVID-19 infection or COVID-19 vaccination. Thus, there is an urgent need to elucidate the effects of COVID-19 and its vaccination on VKH disease.

If COVID-19 and its vaccination have an effect on the development of VKH disease, the total number of patients with VKH disease should increase. In this study, we compared the total number of patients newly diagnosed with VKH disease before and after the declaration of the state of emergency.

## Results

Table 1 shows the demographics of the patients in the Before and After groups. The ratio of newly diagnosed patients with VKH disease normalized by new patients (*P* < 0.05) and patients newly diagnosed with VKH disease normalized by newly diagnosed patients with acute angle closure (AAC) (*P* < 0.001) was significantly higher in the After group than in the Before group. We found no significant differences between the two groups in gender or age. Thirty-eight patients were vaccinated against COVID-19, seven patients did not receive the vaccine, and eight patients were not asked about the vaccine.

**Table 1:** Patient’s demography for Before and After groups.

	Before group (n = 73)	After group (n = 53)	*P* value
Newly diagnosed VKH disease/new patients	73/30,122	53/14,845	< 0.05
Newly diagnosed VKH disease/newly diagnosed patients with AAC	73/58	53/12	< 0.001
Gender (male/female)	31/42	22/31	0.91
Age (years)	48.8 ± 15.4	46.9 ± 17.7	0.53
History of COVID-19 vaccine (yes/no/unknown)		38/7/8	n/a

*AAC* acute angle closure, *VKH* Vogt–Koyanagi–Harada.

Table 2 shows the clinical characteristics of 59 patients who were observed for > 12 months after receiving pulse steroid therapy. We noted no significant differences between both groups in all factors, including body weight at the initial visit, from onset to the initial visit, synechia between the iris and lens at the initial visit, HbA1c at the initial visit, type of VKH disease, fundus appearance at 12 months, recurrence within 12 months, recurrence in non–sunset glow fundus (SGF), recurrence in mild SGF, and recurrence in SGF.

**Table 2:** Characteristics of 59 patients who were observed for > 12 months after pulse steroid therapy in the Before and After groups.

	Before group (n = 32)	After group (n = 27)	*P* value
Body weight (kg)	62.0 ± 15.6	65.0 ± 13.5	0.45
From onset to initial visit (days)	18.4 ± 43.8	9.6 ± 9.8	0.27
Synechia between iris and lens (yes, no)	1, 31	5, 22	0.051
HbA1c (%)	6.1 ± 1.4	5.5 ± 0.4	0.075
Type (complete, incomplete, probable)	16, 15, 1	9, 13, 5	0.11
Fundus appearance at 12 months (non SGF, mild SGF, SGF)	13, 11, 8	16, 8, 3	0.27
Recurrence within 12 months (yes, no)	8, 24	6, 21	0.89
Recurrence in non SGF (yes, no)	1, 12	0, 16	0.26
Recurrence in mild SGF (yes, no)	2, 9	3, 5	0.35
Recurrence in SGF (yes, no)	5, 3	3, 0	0.21

*SGF* sunset glow fundus.

Table 3 shows the fundus condition of 59 patients who were observed for > 12 months after pulse steroid therapy at the initial visit. No significant differences were observed in intraretinal cyst or subretinal fibrinous exudate between the groups.

**Table 3:** Fundus condition of 59 patients who were observed for > 12 months after pulse steroid therapy in the Before and After groups at the initial visit.

	Before group (n = 64 eyes)	After group (n = 54 eyes)	*P* value
Intraretinal cyst (yes, no)	30, 34	23, 31	0.64
Subretinal fibrinous exudate (yes, no)	27, 37	14, 40	0.065

We observed bilateral serous retinal detachments in 40 patients: four had serous retinal detachment in the unilateral eye but no specific changes in the other eye, one had serous retinal detachment in the bilateral eyes and choroidal detachment in one eye, one exhibited serous retinal detachment in one eye and retinal pigment undulation in the other eye, six had bilateral optic disk edema, two had optic disk edema in one eye but no specific changes in the other eye, three had bilateral serous retinal detachments with optic disk edema, one had serous retinal detachments with optic disk edema in the unilateral eye but no specific changes in the other eye, and one had bilateral retinal pigment undulation (Table 4).

**Table 4:** Fundus condition of 59 patients who were observed for > 12 months after pulse steroid therapy in the Before and After groups.

	Before group (n = 32)	After group (n = 27)
Bilateral SRDs (patients)	24	16
Bilateral SRDs, unilateral CD (patient)	1	0
SRD in one eye, NSCs in TOE (patients)	2	2
SRD with DE in one eye, NSCs in TOE (patient)	0	1
SRD in one eye, RPU in TOE (patient)	1	0
Bilateral DE (patients)	2	4
DE in one eye, NSCs in TOE (patients)	2	0
Bilateral SRD with DE (patients)	0	3
Bilateral RPUs (patient)	0	1

*CD* choroidal detachment, *DE* disk edema, *NSC* no specific change, *RPU* retinal pigment undulation, *SRD* serous retinal detachment, *TOE* the other eye, *VKHD* Vogt–Koyanagi–Harada disease.

**Figure 1:**
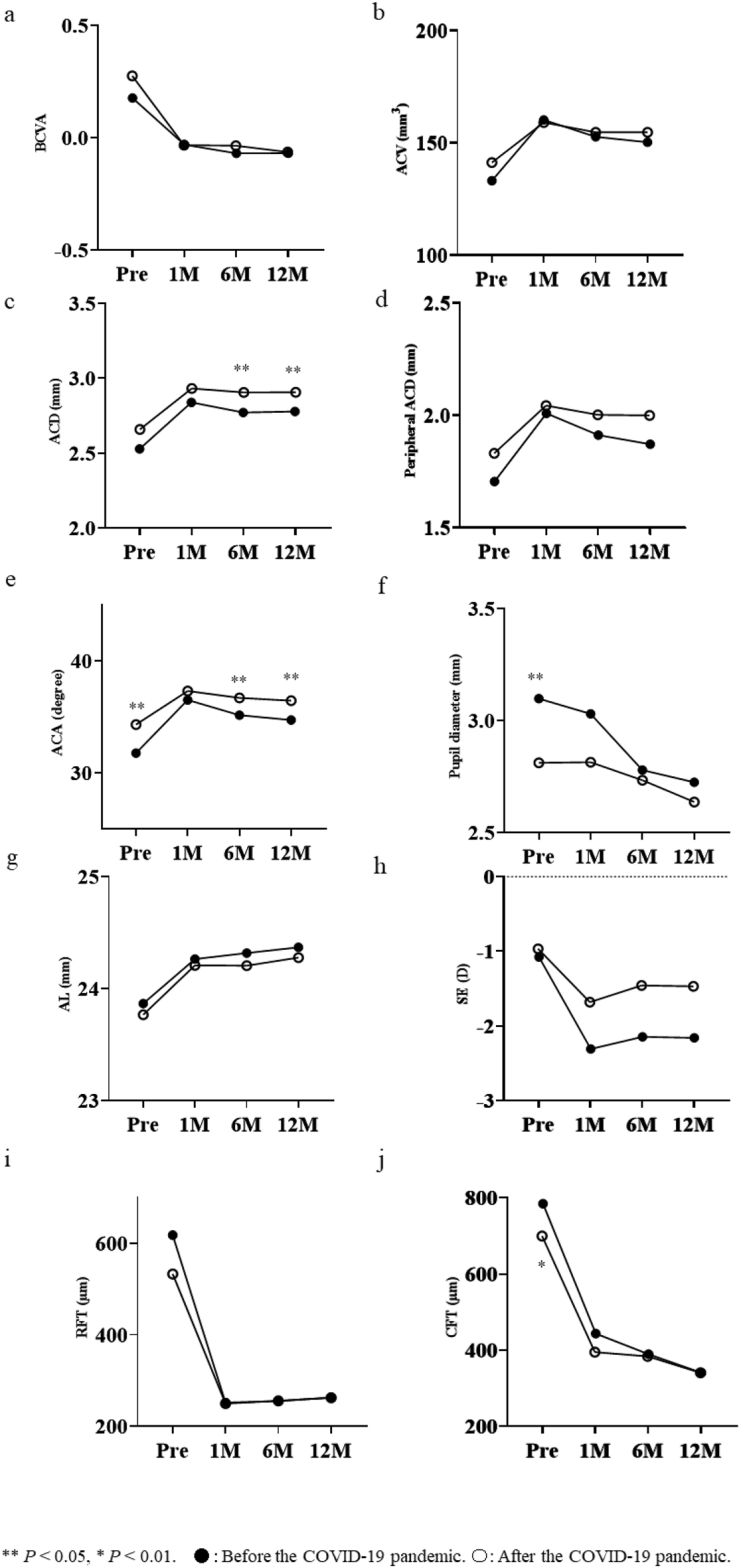
(**a**) Longitudinal changes in the best-corrected visual acuity (BCVA) as the logarithm of the minimal angle of resolution for both the Before and After groups without a significant difference between the groups. (**b**) The anterior chamber volume at pretreatment and months 1, 6, and 12 without significant difference. (**c**) The anterior chamber depth at pretreatment and months 1, 6, and 12 with significant differences at months 6 and 12. (**d**) The peripheral anterior chamber depth at pretreatment and months 1, 6, and 12 without significant difference. (**e**) The anterior chamber angles at the pretreatment and months 1, 6, and 12 with significant differences at pretreatment and months 6 and 12. (**f**) The pupil diameter at the pretreatment and months 1, 6, and 12 with significant differences at pretreatment. (**g**) The axial length at pretreatment and months 1, 6, and 12 without significant difference. (**h**) The spherical equivalent at pretreatment and months 1, 6, and 12 without significant difference. (**i**) The retinal foveal thickness at pretreatment and months 1, 6, and 12 without significant difference. (**j**) The choroidal foveal thickness at pretreatment and months 1, 6, and 12 with significant difference at pretreatment. *ACA* anterior chamber angle, *ACD* anterior chamber depth, *ACV* anterior chamber volume, *AL* axial length, *BCVA* best-corrected visual acuity, *CFT* choroidal foveal thickness, *MAR* minimal angle of resolution, *RFT* retinal foveal thickness, *SE* spherical equivalent.

Figure 1 shows the chronological changes in the average values of BCVA, biomarkers representing the anterior chamber structures, axial length (AL), spherical equivalent (SE), retinal foveal thickness (RFT), and choroidal foveal thickness (CFT) for the Before and After groups. We found significant differences between the Before and After groups in anterior chamber depth (ACD) (Fig. 1c) at months 6 (*P* < 0.05) and 12 (*P* < 0.05). We also found significant differences between the groups in the anterior chamber angle (ACA) (Fig. 1e) at pretreatment (*P* < 0.05) and months 6 (*P* < 0.05) and 12 (*P* < 0.05). At pretreatment, we noted significant differences between the groups in pupil diameter (Fig. 1f) and average CFT (Fig. 1j) (*P* < 0.05 and *P* < 0.01, respectively). We observed no significant differences in the average values of BCVA (Fig. 1a), anterior chamber volume (ACV) (Fig. 1b), peripheral ACD (Fig. 1d), AL (Fig. 1g), SE (Fig. 1h) and RFT (Fig. 1i) between the Before and After groups.

**Figure 2:**
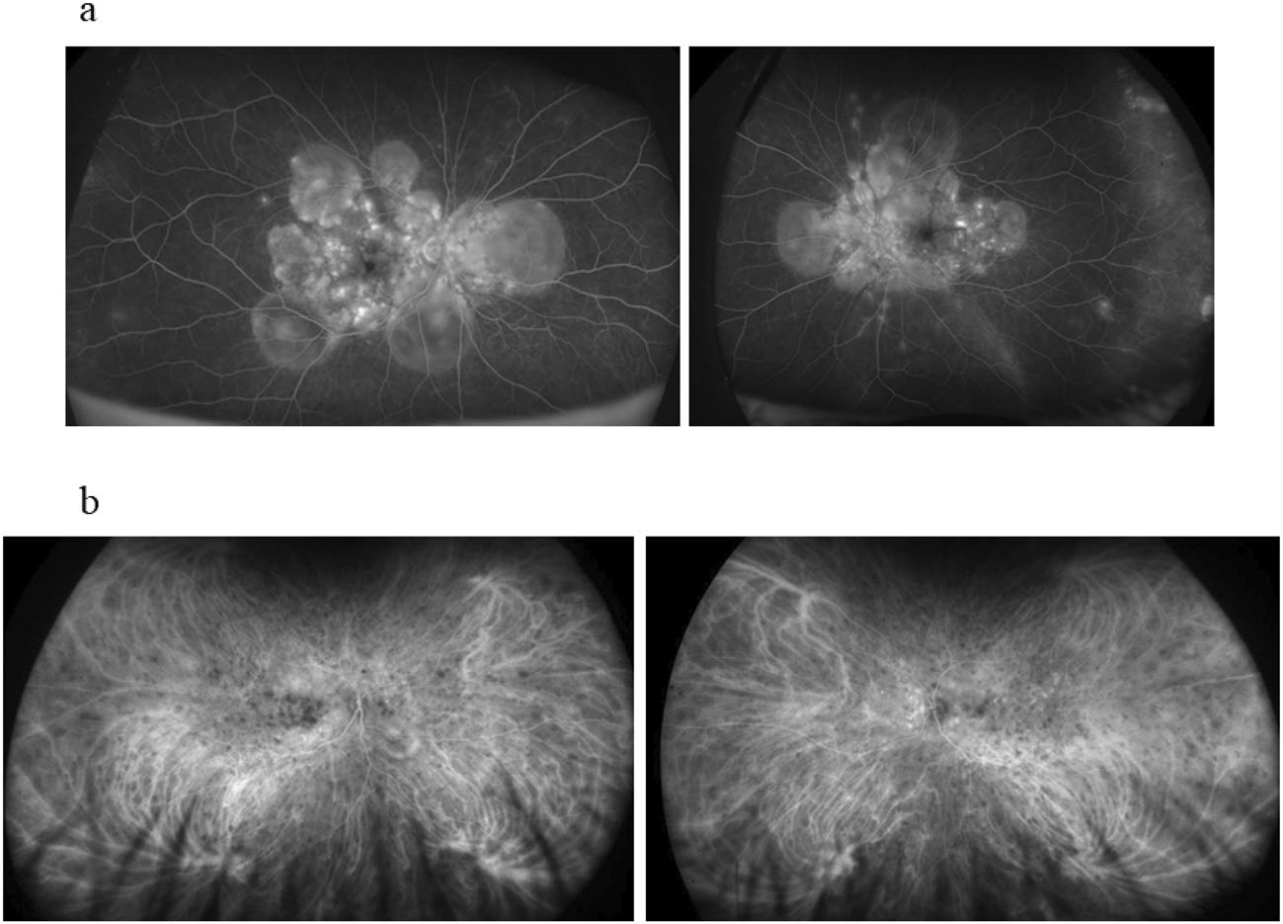
(**a**) Bilateral subretinal dye pooling was observed on fluorescein angiography. (**b**) Many hypofluorescent dark dots and a fuzzy vascular pattern of large stromal vessels were noted in the vascular arcade area on indocyanine green angiography.

Figure 2 shows the fluorescein angiography (FAG) and indocyanine green angiography (ICGA) images of a 56-year old male patient. We used these images to assist in the diagnosis.

**Figure 3:**
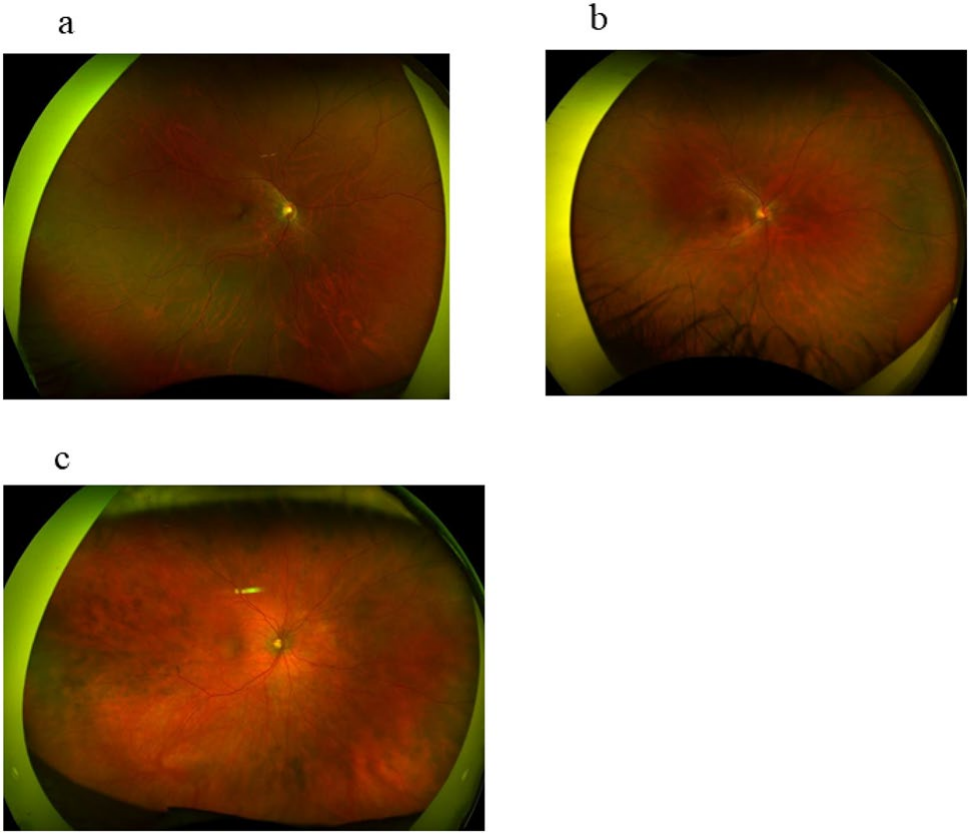
(**a**) Photo of non-sunset glow fundus at 12 months after pulse steroid therapy. There was little difference between the normal retina and non-sunset glow fundus. (**b**) Photo of mild sunset glow fundus at 12 months after pulse steroid therapy. Several choroidal vessels were translucent because of mild depigmentation in the retinal pigment epithelium and choroid. Translucent vessels in the choroid. (**c**) Photo of sunset glow fundus 12 months after pulse steroid therapy. Because many of the choroidal vessels were translucent, the color tone became vermilion.

Figure 3 shows representative fundus images of non-SGF (Fig. 3a), mild SGF (Fig. 3b), and SGF (Fig. 3c) 12 months after pulse steroid therapy. There was a higher rate of translucent vessels in the choroid in SGF than in non-SGF and mild SGF.

## Discussion

Several studies have reported on the development of VKH disease following COVID-19 infection or its vaccination^13–15,17–19^, suggesting that COVID-19 infection and its vaccination might be triggers for the development of VKH disease. A number of articles have reported an association between the development of VKH disease and viral infection^20–23^ or vaccination^24–26^. These reports raise the possibility that the COVID-19 pandemic increased the prevalence of VKH disease due to COVID-19 infection or its vaccination. Counting patients with newly diagnosed VKH disease enables us to understand the prevalence of the disease by analogy. The rate of newly diagnosed VKH disease normalized by new patients increased significantly after the COVID-19 pandemic. However, we considered counting only newly diagnosed VKH to be insufficient, because patients with mild diseases might have avoided visiting the hospital during the COVID-19 pandemic, possibly leading to an overestimation of the prevalence rate of VKH disease. Thus, we selected AAC as a reference disease for the following reasons. First, because AAC acutely and severely affects vision, patients with AAC unexceptionally visit the hospital. Second, structural alternations in the anterior segment of the eye cause AAC, which is unlikely to be affected by COVID-19 infection or vaccination. Even after normalization by the number of newly diagnosed AAC patients, VKH disease prevalence significantly increased after the COVID-19 pandemic, indicating that the COVID-19 pandemic is a possible inducement for the development of VKH disease.

In their study, Manni et al. found no differences in the epidemiology or clinical findings of VKH disease between the COVID-19 and no-COVID-19 eras^27^. Furthermore, they also reported that all patients promptly responded to systemic and local corticosteroid therapy with a good final visual prognosis^27^. There were no significant differences with respect to important clinical features, such as visual prognosis, recurrence rates, SGF rates at 12 months, or type of VKH disease between before and after the declaration of a state of emergency. We found significant differences between the groups in the anterior chamber structures or CFT at some time points after treatment, which indicates that COVID-19 infection or vaccination could modify the recovery process or treatment response. However, previous reports do not support this hypothesis. Lauermann et al. reported that no SARS-CoV-2 was detected in the intraocular fluids of a 72-year-old patient who died of COVID-19 pneumonia^28^. Oren and Kocabas reported that COVID-19 did not affect ACD or pupil diameter^29^. Firat and Kobat and Gokmen and Ozgur reported that CFT and RFT were not affected in patients with recent mild COVID-19 infection^30,31^. Thus, to determine whether COVID-19 infection or vaccination modify the clinical features of VKH disease, further study is needed.

Previous studies have suggested an association between other ocular diseases and the COVID-19 pandemic^32^. Sakamoto et al. showed that the incidence of postvitrectomy endophthalmitis was significantly higher during the COVID mask period. They speculated that, because many people wore masks during the COVID-19 pandemic, oral bacteria were scattered on the ocular surface by breath^32^. Lulla et al. demonstrated that among medical students, the odds ratio dry eye was 1.7 times greater during the pandemic than before the pandemic^33^. They deduced that the instability of tear film was due to the reduced blink rate resulting from prolonged screen time. Lima-Fontes et al. found that the culture-positivity rate of infectious keratitis was significantly higher than that in the prepandemic (2009–2018) data^34^. The authors reported that these changes could be linked to the use of face masks^34^. Fontes Junior et al. reported a decrease in new patients during the COVID-19 pandemic, whereas the rate of malignant tumors increased^35^. On the other hand, unexpected results were reported with regard to retinal vascular occlusive diseases that may have a deep connection with COVID-19^36^. Parks et al. showed that the incidence of retinal artery occlusion and retinal vascular occlusion was rare within 60 days after COVID-19 diagnosis or vaccination^36^. Furthermore, they reported no increase in the hazard ratios of retinal artery occlusion and retinal vein occlusion relative to COVID-19 infection or vaccination, except for a possible increase in the hazard ratio of retinal artery occlusion in women who received the mRNA-1273 vaccine^36^. COVID-19 causes endothelial dysfunction, and elevations were observed in von Willebrand factor antigen and Willebrand factor activity and factor VIII, leading to the activation of the coagulating system and platelet aggregation^37^. Thus, an increase in retinal vascular occlusive diseases was estimated. Further studies are needed to determine the correlation between ocular disease and the COVID-19 pandemic.

The strength of this study is that the total number of patients exceeded 100. Because there are few reports with more than 100 cases of VKH disease^38,39^, this study is significant. Furthermore, we used integrated treatment methods to analyze BCVA, anterior chamber structure, AL, SE, RFT, and CFT in the Before and After groups, which is also an important aspect of this study. We predict that accurate data regarding the connection between VKH disease and the COVID-19 pandemic, as examined in this study, will not be released in the future. No reports examined the BCVA, anterior chamber structure, AL, and SE chronologically before the COVID-19 pandemic, and it is not possible to return to the pre-COVID-19 period to examine these factors. The limitation of this study is that we could not assess the reason for the significant differences in the anterior chamber structures or CFT at some time points after treatment between the Before and After groups. These differences might be key in the future. Although we were able to show that the rate of VKH disease onset increased, we could not clearly determine how COVID-19 and its vaccine affect the onset mechanism of VKH disease. Because the elucidation of the onset mechanism of VKH disease is of primary importance, early clarification is expected.

In conclusion, our results demonstrate that the prevalence of VKH disease increased during the COVID-19 pandemic, indicating that COVID-19 viral infection or vaccination could trigger the development of VKH disease. However, it is unlikely that the clinical features of VKH disease were affected by the COVID-19 pandemic.

## Methods

### Patients

In this study, we retrospectively evaluated 126 patients with acute VKH disease who visited Dokkyo Medical University Saitama Medical Center and Fukushima Medical University between April 2015 and December 2022. We followed the acute uveitic phase of VKH disease referred to in previous reports^40,41^. During the same period, 44,967 new patients and 70 patients with AAC were similarly evaluated. We categorized new patients, patients with newly diagnosed VKH disease, and patients with AAC into “Before” and “After” groups based on the initial visit and the declaration of the state of emergency. Fifty-nine patients who were observed for > 12 months after pulse steroid therapy were also divided into Before and After groups and compared in detail. The type and diagnostic criteria of VKH disease were followed by the newly revised diagnostic criteria^42^.

In this study, we excluded patients with recurrent/chronic VKH disease, senile dementia, ischemic optic neuropathy, and myopia with more than − 9 diopters or hypermetropia of more than 5 diopters at the initial visit.

Steroid therapy was initiated by infusing methylprednisolone (Solu-Medrol; Pfizer, New York, NY, USA) at 1000 mg/day for 3 days, followed by oral administration of prednisolone (Predonine, Shionogi, Osaka, Japan). Oral administration of prednisolone was started at 40 mg/day, and the dose was tapered by 5 mg/day every 3 to 4 weeks.

This study adhered to the tenets of the Declaration of Helsinki. The Institutional Review Board of Dokkyo Medical University Saitama Medical Center and Fukushima Medical University approved the study protocol. Due to retrospective nature of the study, the need for informed consent of patients was waived by the Ethics Committee of Dokkyo Medical University Saitama Medical Center and Fukushima Medical University.

### Measurement of the biomarkers

At the first visit, all patients underwent comprehensive ophthalmologic evaluations, including standardized refraction and measurement of BCVA using a Landolt ring, slit-lamp biomicroscopy, optical coherence tomography (OCT), and color fundus photography.

For diagnosis, 110 patients underwent FAG (Fig. 2a) and indocyanine green angiography ICGA (Fig. 2b); 8 patients did not undergo these examinations because they had recent records of angiographies at the previous hospitals. Four patients could not undergo urgent FAG and ICGA because of the possibility of pregnancy or iodine allergy. The remaining four patient could not undergo these evaluations because of emergency admission. Fifty-nine patients who were observed for > 12 months after pulse steroid therapy underwent the above ophthalmologic examinations, anterior segment analysis, and AL measurement, as described previously^43^. Swept-source OCT (SS-OCT) (PLEX Elite 9000, Carl Zeiss Meditec AG, Jena, Germany) was performed to determine CFT by manually measuring the distance from the outer border of the retinal pigment epithelium to the inner border of the sclera at the fovea. One of the authors (M.S.) independently evaluated the OCT images in a blinded fashion, not knowing the clinical information of any patients. A CFT exceeding > 800 μm was defined as 800 μm because the inner scleral border could not fit in the SS-OCT images. These biomarkers were measured before and months 1, 6, and 12 after the initiation of pulse steroid therapy.

The eyes were divided into three groups according to the SGF condition at 12 months (i.e., SGF group, mild SGF group, and non-SGF group) (Fig. 3a–c). The condition of SGF in each eye was determined by first author (T.M.) by directly examining the fundus of the patients.

We compared these biomarkers between 64 eyes from 32 patients in the Before group and 54 eyes from 27 patients in the After group. We then analyzed the differences in ocular biomarkers in both groups.

### Statistical analysis

Data are expressed as means ± standard deviations. We used the Chi-square test to determine the statistical significance of prevalence rates, sex ratio, synechia between the iris and lens, type of VKH disease, fundus condition at 12 months posttreatment, and recurrence rates. We used an unpaired *t*-test to compare the groups before and after the declaration of a state of emergency. StatMate version V for Macintosh (ATMS, Tokyo, Japan) was used in these analyses. We considered differences with a *P* value of < 0.05 to be statistically significant.

## Data Availability

The data sets used and/or analyzed during the current study are available from the corresponding author on reasonable request.
